# Novel Combination of COX-2 Inhibitor and Antioxidant Therapy for Modulating Oxidative Stress Associated with Intestinal Ischemic Reperfusion Injury and Endotoxemia

**DOI:** 10.3390/antiox9100930

**Published:** 2020-09-28

**Authors:** Enrico Gugliandolo, Marika Cordaro, Rosalba Siracusa, Ramona D’Amico, Alessio Filippo Peritore, Tiziana Genovese, Daniela Impellizzeri, Rosanna Di Paola, Rosalia Crupi, Salvatore Cuzzocrea, Roberta Fusco

**Affiliations:** 1 Department of Chemical, Biological, Pharmaceutical and Environmental Sciences, University of Messina, 98166 Messina, Italy; egugliandolo@unime.it (E.G.); rsiracusa@unime.it (R.S.); rdamico@unime.it (R.D.); aperitore@unime.it (A.F.P.); tgenovese@unime.it (T.G.); dimpellizzeri@unime.it (D.I.); dipaolar@unime.it (R.D.P.); rfusco@unime.it (R.F.); 2 Department of Biomedical, Dental and Morphological and Functional Imaging, University of Messina, Via Consolare Valeria, 98125 Messina, Italy; cordarom@unime.it; 3 Department of Veterinary Sciences, University of Messina, 98168 Messina, Italy; 4 Department of Pharmacological and Physiological Science, Saint Louis University School of Medicine, Saint Louis, MO 63104, USA

**Keywords:** intestinal strangulation, antioxidant

## Abstract

Background: Intestinal ischemic reperfusion (I/R) injury is associated with a high mortality rate; this condition is also related to significant endotoxemia and systemic inflammation. The preservation of tissue perfusion and a sufficient blood flow are required to deliver nutrients and oxygen, preserve metabolic pathways, and eliminate waste products. Oxidative stress plays a fundamental role in intestinal I/R injury and leads to disruption of the mucosal barrier and necrosis, allowing the migration of endotoxins and luminal bacteria into the systemic circulation. In this study, we evaluated the beneficial effects of a cyclooxygenase (COX)-2 inhibitor—firocoxib—plus the antioxidant vitamin C in a rat model of intestinal I/R injury. Methods: We used a rat model of I/R injury in which the superior mesenteric artery was clamped for 30 min by a vascular clamp, and the animals were then allowed 1 h of reperfusion. Results: Our results show the importance of combined anti-inflammatory and antioxidant treatment for the prevention of intestinal I/R injury that leads to reduced systemic endotoxemia. We observed a significantly synergistic effect of firocoxib and vitamin C in reducing intestinal wall damage and oxidative stress, leading to a significant reduction of inflammation and endotoxemia. Conclusions: Our results indicate that this approach could be a new pharmacological protocol for intestinal colic or ischemic injury-induced endotoxemia.

## 1. Introduction

Intestinal obstruction has been defined as simultaneous vascular and luminal damage that can be of ischemic or hemorrhagic origin [1]. It leads to disruption of the intestinal barrier function, hypovolemia, endotoxemia, and cytotoxic shock [2]. In particular, ischemic strangulating obstruction comprises simultaneous occlusion of the arterial vasculature and intestinal venous vasculature, resulting in cyanotic and blanched serosa [1]. The duration and magnitude of the reduced blood flow determine the severity of ischemic tissue. Most tissues can resist a considerable decrease in blood flow due to their ability to increase oxygen extraction and stock cellular energy [3]. Restoration of the oxygen supply and blood flow in ischemic tissues is required to re-establish a regular function and repair impaired components. Small intestine and large colon obstructions are common causes of acute abdominal disease in horses. Small intestine obstruction frequently results from either a volvulus or incarceration at several intraabdominal locations. The pathophysiological processes associated with ischemia and reperfusion (I/R) likely occur in the intestinal tract of horses during and after the surgical correction of naturally acquired intestinal vascular compromise. Intestinal ischemic injury in horses is associated with a high mortality rate—50% to 80%. The prognosis depends on the degree and duration of ischemia before surgical intervention. Despite surgical correction, intensive medical therapy, and supportive care, the perioperative mortality is high. This is likely a reflection of the severe damage the intestine sustains during the ischemic period and the further injury that occurs upon reperfusion. The progressive mucosal epithelial damage induces a disruption of the mucosal barrier and necrosis, allowing the migration of endotoxins and luminal bacteria into the systemic circulation. 

Nonsteroidal anti-inflammatory drugs (NSAIDs) work through the inhibition of cyclooxygenase (COX) and are highly effective for the treatment of pain and inflammation in horses, reducing local prostaglandin (PG) synthesis and inflammatory mediators. While COX-1 is constitutively expressed in the equine intestine and is responsible for PG synthesis in normal conditions [4], COX-2 is upregulated in response to injury. Firocoxib is a COX-2-selective NSAID extensively used in horses, although the use of NSAIDs in the pharmacological treatment of clinical conditions due to damage from ischemia reperfusion has not been completely elucidated [5,6,7]. In particular, several studies have proven the superior efficacy of firocoxib in the treatment of intestinal ischemia in horses compared to flumixine meglumine [8]. During I/R injury and related cellular damage, oxidative stress also plays a key role. The restoration of tissue oxygenation, however, is followed by biochemical changes that can lead to an increased generation of reactive oxygen species (ROS) and be fatal to tissues. In particular, in the small intestine of horses, during complete arteriovenous ischemia, ROS production is responsible for lipid peroxidation. A decrease in the activity of superoxide dismutase (SOD)—an endogenous anti-oxidative enzyme—accompanies an increased tissue malondialdehyde (MDA) concentration during ischemia, potentially predisposing the intestinal mucosa to I/R damage. Several previous studies from our group have described the beneficial effects of natural antioxidant treatments in different pathologies [9,10,11,12,13,14,15,16,17]. Unfortunately, firocoxib did not display any antioxidant activity. On the other hand, the efficacy of the antioxidant vitamin C in restoring the oxidative balance is well-known [18,19].

Therefore, in line with the available evidence, the aim of the present study was to combine the COX-2 inhibitor firocoxib and the antioxidant vitamin C to fight the oxidative stress that characterizes intestinal I/R injury in horses using a rat system as a model.

## 2. Materials and Methods 

### 2.1. Animals

Male rats (Sprague–Dawley, 200–230 g; Envigo, Milan, Italy) were employed. The University of Messina Review Board for Animal Care (OPBA) approved the study. All animal experiments complied with the new Italian regulations (D.Lgs 2014/26), EU regulations (EU Directive 2010/63), and ARRIVE guidelines.

### 2.2. Experimental Protocol

Rats were randomly divided into the following groups (*n* = 10): Firocoxib (10 mg/kg), vitamin C (750 mg/kg) alone, or firocoxib (10 mg/kg) in combination with vitamin C (750 mg/kg), administered intraperitoneally 30 min before I/R induction [19,20,21,22]. Animals were anesthetized with sodium pentobarbital (45 mg/kg) and the induction of I/R was performed as previously described [23]. Briefly, the superior mesenteric artery was occluded for 30 min with a vascular clamp, and the animals were then allowed 1 h of reperfusion after clamp removal. After this time, blood was collected by intra-cardiac puncture and animals were sacrificed by cervical dislocation under anesthesia. Ileum tissue samples were collected for histological and biochemical analyses. In another set of experiments, following reperfusion, the various groups of animals were observed for 4 h to evaluate survival differences [24].

### 2.3. Experimental Groups

(1) I/R + vehicle (saline): Rats were subjected to surgery and treated with a vehicle.
(2) I/R + firocoxib (10 mg/kg): Rats were subjected to surgery and treated with firocoxib (10 mg/kg).
(3) I/R + vitamin C (750 mg/kg): Rats were subjected to surgery and treated with vitamin C (750 mg/kg).
(4) I/R + firocoxib (10 mg/kg) + vitamin C (750 mg/kg): Rats were subjected to surgery and treated with firocoxib (10 mg/kg) + vitamin C (750 mg/kg).
(5) Sham groups: Animals were operated on with surgical steps; however, they were not subjected to I/R and were treated with either a vehicle, firocoxib alone, vitamin C alone, or firocoxib + vitamin C.

### 2.4. Measurement of Lipid Peroxidation

Lipid peroxidation was evaluated by a reaction between malondialdehyde (MDA), thiobarbituric acid, and lipid peroxides and measured spectrophometrically at 532 nm [13]. The results were expressed as nanomoles of thiobarbituric acid (TBA) reactant formed per gram of wet tissue.

### 2.5. Myeloperoxidase Activity 

Myeloperoxidase (MPO) activity, which is an indicator of polymorphonuclear leukocytes (PMN) accumulation, was determined spectrophotometrically at 650 nm [25]. MPO activity was expressed in U per gram weight of wet tissue and defined as the quantity of enzyme degrading 1 µmol of peroxide min^−1^ at 37 °C [26].

### 2.6. Measurement of the Protein Carbonyl Content 

The protein carbonyl content (PCC) was evaluated spectrophotometrically at 370 nm by the reaction between 2,4-dinitrophenylhydrazine and the carbonyl group [27]. The results were expressed as nanomoles of carbonyl per milligram of protein. 

### 2.7. Determination of Antioxidant Enzyme Activity

Ileum tissues were homogenized, and the supernatant collected for the determination of total glutathione (Glutathione Assay Kit; Trevigen Inc., Gaithersburg, MD, USA). The results of glutathione (GSH) levels were expressed as nmol/mg of protein [23]. SOD activity was determined on ileum tissue homogenate according to the nitro blue tetrazolium reduction assay [28]. Superoxide dismutase (SOD) activity was expressed as U/mg of protein. Catalase (CAT) activity was estimated by a decreased absorbance of H_2_O_2_ at 240 nm [29]. Glutathione peroxidase (GPx) activity was determined as described [23]. Oxidized glutathione (GSSG) is reduced by glutathione reductase and NADPH. The oxidation of NADPH to NADP+ was evaluated by a decreased absorbance at 340 nm, and GPx activity was expressed as U/mg of protein. Glutathione-S-transferase (GST) activity was determined spectrophotometrically at 340 nm [23]. For this measurement, 1 U is equal to the amount of enzyme producing 1 mmol of CDNB-GSH conjugate per minute.

### 2.8. Evaluation of PGE2, Tumor Necrosis Factor Alpha (TNF-α), Interleukin (IL)-6, IL-1β, D-Lactate, Diamine Oxidase (DAO), and Endotoxin Levels

Serum interleukin IL-1β and IL-6 and tumor necrosis factor alpha (TNF-α) levels were determined using an ELISA kit (R&D Systems Inc., Minneapolis, MN, USA) [30]. Serum levels of D-lactate and DAO were investigated spectrophotometrically (Sigma-Aldrich, St. Louis, MO, USA; Merck KGaA). Serum levels of endotoxin were determined by a Limulus Amebocyte Lysate Assay kit (Shanghai Biochemical Co., Ltd., Shanghai, China) [31]. PGE2 ileal tissue expression was determined according to the manufacturer’s instructions (MyBiosource, Bergamo, Italy) [32].

### 2.9. Histological Examination

Ileum tissues were collected after 1 h of reperfusion. After fixing the tissues in buffered formaldehyde solution (10% in phosphate buffered saline (PBS)), histological sections were stained with hematoxylin and eosin and evaluated using a Leica DM6 microscope (Leica Microsystems SpA, Milan, Italy) associated with Leica LAS X Navigator software (Leica Microsystems SpA, Milan, Italy). The morphological criteria were considered as already described [33]: 0, no damage; 1 (mild), focal epithelial edema and necrosis; 2 (moderate), diffuse swelling and necrosis of the villi; 3 (severe), necrosis with the presence of neutrophil infiltrate in the submucosa; and 4 (highly severe), widespread necrosis with massive neutrophil infiltrate and hemorrhage. All images were acquired at 10× magnification (250 μm). 

### 2.10. Bacterial Translocation

Mesenteric lymph nodes (MLNs) and caudal lymph nodes (CLNs) were harvested for bacteriological analysis [34]. In addition, blood underwent bacterial colony counts [35].

### 2.11. Western Blot Analysis

Western blots were performed as described in our previous studies [14,36]. Specific primary antibody anti-COX-2 (1:600, Santa Cruz Biotechnology) or anti-PGE2 (1:700; Bioss Antibodies) was mixed in 1× PBS, 5% *w*/*v* nonfat dried milk, and 0.1% Tween-20, and incubated at 4 °C, overnight. After that, blots were incubated with the peroxidase-conjugated bovine anti-mouse IgG secondary antibody or peroxidase-conjugated goat anti-rabbit IgG (1:2000, Jackson Immuno Research) for 1 h at room temperature. To verify the equal amounts of protein, membranes were also incubated with the antibody against beta actin (1:1000; Santa Cruz Biotechnology). Signals were detected with enhanced chemiluminescence detection system reagent (Super-Signal West Pico Chemiluminescent Substrate, Pierce). The relative expression of protein bands was quantified by densitometry with Bio-Rad ChemiDoc XRS software and standardized to β-actin levels. Images of blot signals were imported to analysis software (Image Quant TL, v2003).

### 2.12. Statistical Evaluation

All values in the figures and text are expressed as the mean ± standard error of the mean (SEM) of N number of animals. The results were analyzed by one-way ANOVA, followed by Bonferroni’s post-hoc test, for multiple comparisons. A *p*-value of <0.05 was considered significant; * *p* < 0.05 vs. sham + vehicle, ^#^ *p* < 0.05 vs. vehicle, ** *p* < 0.01 vs. sham + vehicle, ^##^ *p* < 0.01 vs. vehicle, *** *p* < 0.001 vs. sham + vehicle, and ^###^ *p* < 0.001 vs. vehicle.

## 3. Results

### 3.1. Firocoxib+Vit C Reduces Lethality and Falls of the Arterial Blood Pressure

**Figure 1:**
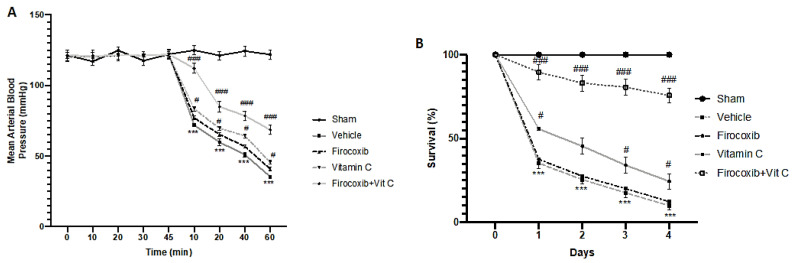
Effect of combined therapy of firocoxib and vitamin C on ischemic reperfusion (I/R)-induced mortality and arterial blood pressure: (**A**) Mean arterial blood pressure and (**B**) survival (%). *n* = 5 animals from each group for each analysis. A *p*-value of <0.05 was considered significant; ^#^ *p* < 0.05 vs. vehicle, *** *p* < 0.001 vs. sham + vehicle, and ^###^ *p* < 0.001 vs. vehicle.

In animals with I/R injury, reperfusion after 30 min of ischemia caused an abrupt and sustained decrease in systemic blood pressure, indicating circulatory shock (Figure 1A, Supplemental Figure S1). An intense shock state is associated with an increased mortality at the end of the reperfusion period (Figure 1B, Supplemental Figure S1). Firocoxib and vitamin C decreased mortality and falling blood pressure. Firocoxib+Vit C treatment significantly reduced mortality (Figure 1B, Supplemental Figure S1) and falling blood pressure (Figure 1A). 

### 3.2. Firocoxib+Vit C Reduces COX-2 and PGE2 Expression

**Figure 2:**
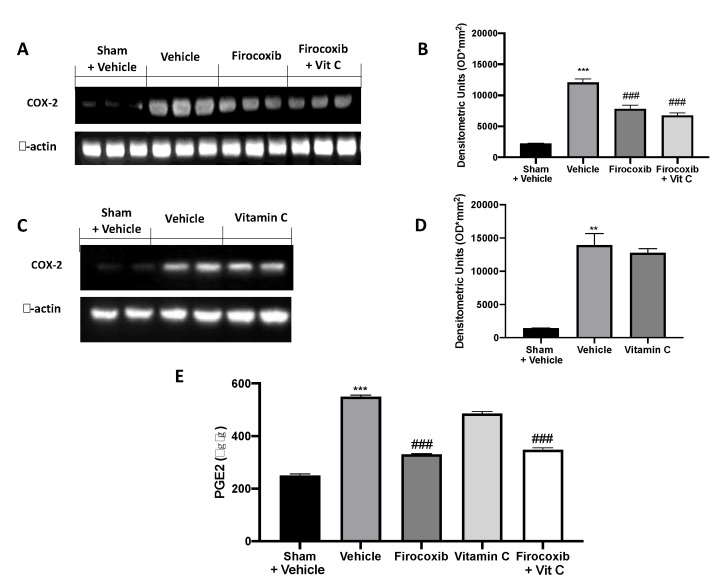
Effect of combined firocoxib and vitamin C therapy on cyclooxygenase (COX)-2 and PGE2 expression: Western blot analysis of (**A**) COX-2, (**B**) densitometric analysis, (**C**) COX-2, (**D**) densitometric analysis, (**E**) PGE2 expression. *n* = 5 animals from each group for each analysis. A *p*-value of <0.05 was considered significant; ** *p* < 0.01 vs. sham + vehicle, *** *p* < 0.001 vs. sham + vehicle, and ^###^ *p* < 0.001 vs. vehicle.

Western blot analysis of ileum tissue showed increased COX-2 expression in vehicle-treated animals compared to the sham + vehicle group (Figure 2A, also see densitometric analysis B, C; see densitometric analysis D, Supplemental Figure S1). Firocoxib and Firocoxib+Vit C decreased COX-2 and PGE2 levels (Figure 2A, see densitometric analysis B, E). Vitamin C treatment did not affect COX-2 expression (Figure 2 C, see densitometric analysis D) and PGE2 levels (Figure 2E).

### 3.3. Firocoxib+Vit C Enhances the Antioxidant/Oxidant Balance during I/R Injury 

**Figure 3:**
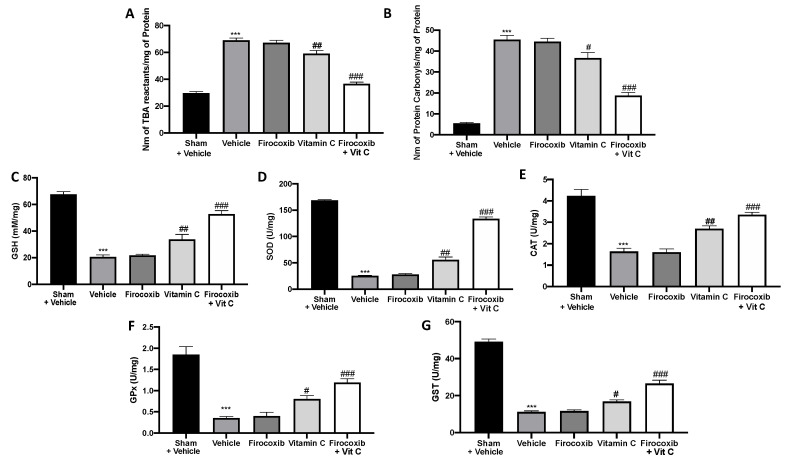
Effect of combined firocoxib and vitamin C therapy on oxidative stress: (**A**) Lipid peroxidation, (**B**) protein carbonyl, (**C**) total glutathione (GSH), (**D**) superoxide dismutase (SOD), (**E**) catalase (CAT), (**F**) glutathione peroxidase (GPx), and (**G**) glutathione-S-transferase (GST). *n* = 5 animals from each group for each analysis. A *p*-value of <0.05 was considered significant; ^#^ *p* < 0.05 vs. vehicle, ^##^ *p* < 0.01 vs. vehicle, *** *p* < 0.001 vs. sham + vehicle, and ^###^ *p* < 0.001 vs. vehicle.

ROS production was one of the earliest mechanisms postulated to explain tissue demise after an ischemic insult. ROS generated by hypoxia or reoxygenation are now recognized to interact with physiological signal transducers rather than to be simple reactants that peroxidize membrane lipids, oxidize DNA, or denature enzyme proteins. Animals treated with the vehicle showed increased lipid peroxidation (Figure 3A) and PCC (Figure 3B). No difference between vehicle-treated and firocoxib-treated rats was detected. Firocoxib+Vit C treatment was more effective in reducing lipid peroxidation (Figure 3A, Figure S1) and PCC (Figure 3B, Figure S1) than vitamin C alone. I/R in vehicle-treated rats decreased the antioxidant enzyme activity of CAT, SOD, GST, GPx, and GSH. Firocoxib+Vit C treatment restored the antioxidant status changed by I/R injury (Figure 3C–G respectively, Figure S1). Firocoxib treatment alone was not able to significantly increase the antioxidant enzyme activity.

### 3.4. Firocoxib+Vit C Modulates MPO Activity, the Cytokine Plasma Level, the Intestinal Barrier Function, and Bacterial Translocation

**Figure 4:**
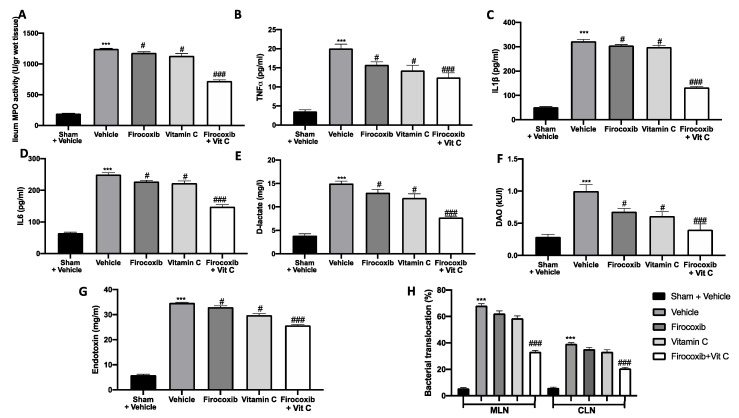
Effect of combined therapy of firocoxib and vitamin C on myeloperoxidase (MPO) activity, tumor necrosis factor alpha (TNF-α), interleukin (IL)-6, IL-1β, D-lactate, diamine oxidase (DAO), and endotoxin levels, and bacterial migration: (**A**) MPO activity, (**B**) serum TNF-α, (**C**) IL-1β, (**D**) IL-6, (**E**) D-lactate, (**F**) DAO, (**G**) endotoxin levels, and (**H**) bacterial translocation. *n* = 5 animals from each group for each analysis. A *p*-value of <0.05 was considered significant; ^#^ *p* < 0.05 vs. vehicle, *** *p* < 0.001 vs. sham + vehicle, and ^###^ *p* < 0.001 vs. vehicle.

The reperfusion of the ischemic mesenteric circulation was also characterized by an increase in MPO activity, which is an indicator of neutrophil accumulation in the ileum. MPO activity was reduced by both treatments (Figure 4A). In all analyzed parameters, the Firocoxib+Vit C treatment displayed major efficacy. Additionally, the inflammatory process is also characterized by an increased expression of pro-inflammatory cytokines. TNF-α, IL-6, and IL-1β plasma levels were increased in vehicle-treated animals (Figure 4B–D). Firocoxib, vitamin C, and Firocoxib+Vit C administration reduced cytokine plasma levels (Figure 4B–D). Additionally, all treatments reduced D-lactate, DAO, and endotoxin serum levels, showing protective effects on the intestinal barrier function (Figure 4E–G). Moreover, vehicle-treated mice showed increased bacterial translocation to mesenteric lymph nodes (MLNs) and caudal lymph nodes (CLNs). Firocoxib, vitamin C, and Firocoxib+Vit C treatment reduced bacterial translocation to MLNs and CLNs (Figure 4H). Firocoxib+Vit C treatment displayed more efficacy than firocoxib alone and vitamin C in reducing cytokine levels, improving the mucosal barrier function and decreasing bacterial translocation.

### 3.5. Firocoxib+Vit C Reduces Histological Alterations Caused by I/R Injury

**Figure 5:**
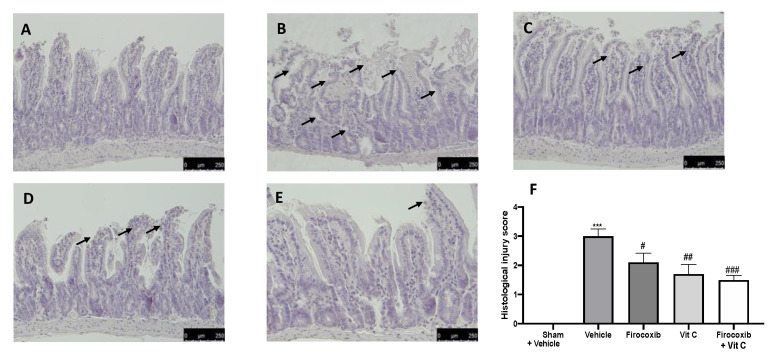
Effect of combined therapy of firocoxib and vitamin C on I/R-induced intestine injury and MPO activity: hematoxylin and eosin (H&E) staining: (**A**) sham + vehicle, (**B**) vehicle, (**C**) firocoxib, (**D**) vitamin C, (**E**) Firocoxib+Vit C, (**F**) histological injury score. *n* = 5 animals from each group for each analysis. A *p*-value of <0.05 was considered significant; ^#^ *p* < 0.05 vs. vehicle, ^##^ *p* < 0.01 vs. vehicle, *** *p* < 0.001 vs. sham + vehicle, and ^###^ *p* < 0.001 vs. vehicle.

Histological examination of the ileum after the I/R injury revealed expected and characteristic pathological changes. Histological features of normal gut tissue were observed in gut tissues prepared from sham rats (Figure 5A,F). The ileum of vehicle-treated animals displayed damage to the villi tips and diffuse inflammatory cell infiltrates in the submucosa (Figure 5B,F). Firocoxib (Figure 5C,F) and vitamin C (Figure 5D,F) and Firocoxib+Vit C showed decreased ileum damage (Figure 5E,F).

## 4. Discussion

The aim of this study was to evaluate the combined treatment of firocoxib—a COX-2 selective inhibitor—and vitamin C—a strong antioxidant molecule—for fighting the inflammation and oxidative stress that characterize intestinal I/R injury, using a rat system as a model. This could be an important pharmacological protocol across different species in the management of clinical conditions such as equine colic. Our results show that, as expected, based on the known mechanism of action of these molecules, firocoxib administration was able to reduce COX-2 expression, while vitamin C did not. The combined administration of both was able to downregulate COX-2 and PGE2 expression. Additionally, this combined therapy increased the endogenous antioxidant systems. Endogenous antioxidant systems such as catalase, superoxide dismutase, and glutathione peroxidase protect against oxidant injury [37]. In order to prevent the secondary generation of hydroxyl radicals, catalase reduces hydrogen peroxide to water. Superoxide dismutase transforms superoxide anions to hydrogen peroxide. Glutathione peroxidase uses glutathione as the substrate to transform hydrogen peroxide to water [38]. Treatment with firocoxib alone did not show any antioxidant activity compared to combined therapy. It is well-described that rats subjected to I/R injury display a significant increase in tissue MPO activity, cytokine levels, and marked injury to the distal ileum [24]. Firocoxib administered in combination with vitamin C was able to reduce small intestine damage and decrease neutrophil accumulation in the injured tissues, as shown by the MPO activity, with more efficacy than the compounds administered alone. The consequences of intestinal I/R injury include an altered absorptive function of the intestine; increased intestinal hyperpermeability with bacterial translocation; and the production of molecules such as hydrogen peroxide, superoxide, endotoxins, and inflammatory cytokines that may harm distant organs [39]. Numerous studies have shown that an increase of both D-lactate and DAO serum levels indicates functional and structural alteration in the intestinal mucosa permeability [40,41,42]. DAO is an enzyme primarily resident in the intestinal villus; its serum expression also increases following impairment to the mucosal barrier function [43]. The increased barrier permeability is also related to increased endotoxin serum levels. Endotoxins are molecules in the walls of bacilli that induce septic shock, sepsis, and gut-derived bacteremia; furthermore, these molecules are able to induce a very harmful inflammatory response known as a “cytokine storm” [44]. The combined administration of firocoxib with vitamin C was able to reduce impairment of the intestinal mucosal barrier, as shown by decreasing D-lactate, DAO, and endotoxin serum levels. Intestinal I/R injury also results in multiorgan failure and the release of several endogenous inflammatory mediators. We detected increased plasma cytokine levels and the translocation of bacteria and toxins in animals subjected to I/R [45]. Firocoxib treatment in combination with vitamin C was able to decrease cytokine plasma expression, toxins, and bacterial migration. From a histological point of view, I/R injury decreases the villus height, mucosal thickness, and crypt depth [46]. The combined administration of firocoxib with vitamin C, by reducing COX-2 expression and the related PG and cytokine production, exhibited an antioxidant capacity and, by reducing neutrophil recruitment to the lesion site, histological protection. 

## 5. Conclusions

The results of our study show the usefulness of pharmacological combination therapy of firocoxib and vitamin C for alleviating intestinal injury due to ischemic reperfusion events. In particular, we show the importance of combined anti-inflammatory and antioxidant treatment for preventing intestinal I/R injury, which leads to reduced systemic endotoxemia. Our results indicate that this pharmacological approach could be a new protocol for managing ischemic conditions and combatting endotoxemia, such as that faced in equine intestinal colic.

## Supplementary Materials

The following are available online at https://www.mdpi.com/2076-3921/9/10/930/s1. Figure S1. Effect of combined therapy of firocoxib and vitamin C on sham animals.
