# Supplementary material for: Novel Combination of COX-2 Inhibitor and Antioxidant Therapy for Modulating Oxidative Stress Associated with Intestinal Ischemic Reperfusion Injury and Endotoxemia
Source: Antioxidants (Basel). 2020 Sep 28;9(10):930. doi: 10.3390/antiox9100930 (PMC7601577; doi:10.3390/antiox9100930)
Supplement: Supplementary file 1 [file antioxidants-09-00930-s001.pdf]

Figure S1. Effect of combined therapy of Firocoxib and Vitamin C on sham animals: Mean arterial blood pressure (A), Survival % (B), Western Blot analysis of COX-2 (C), Densitometric analysis (C’), total glutathione (GSH)(D), superoxide dismutase (SOD) (E), catalase (CAT) (F).

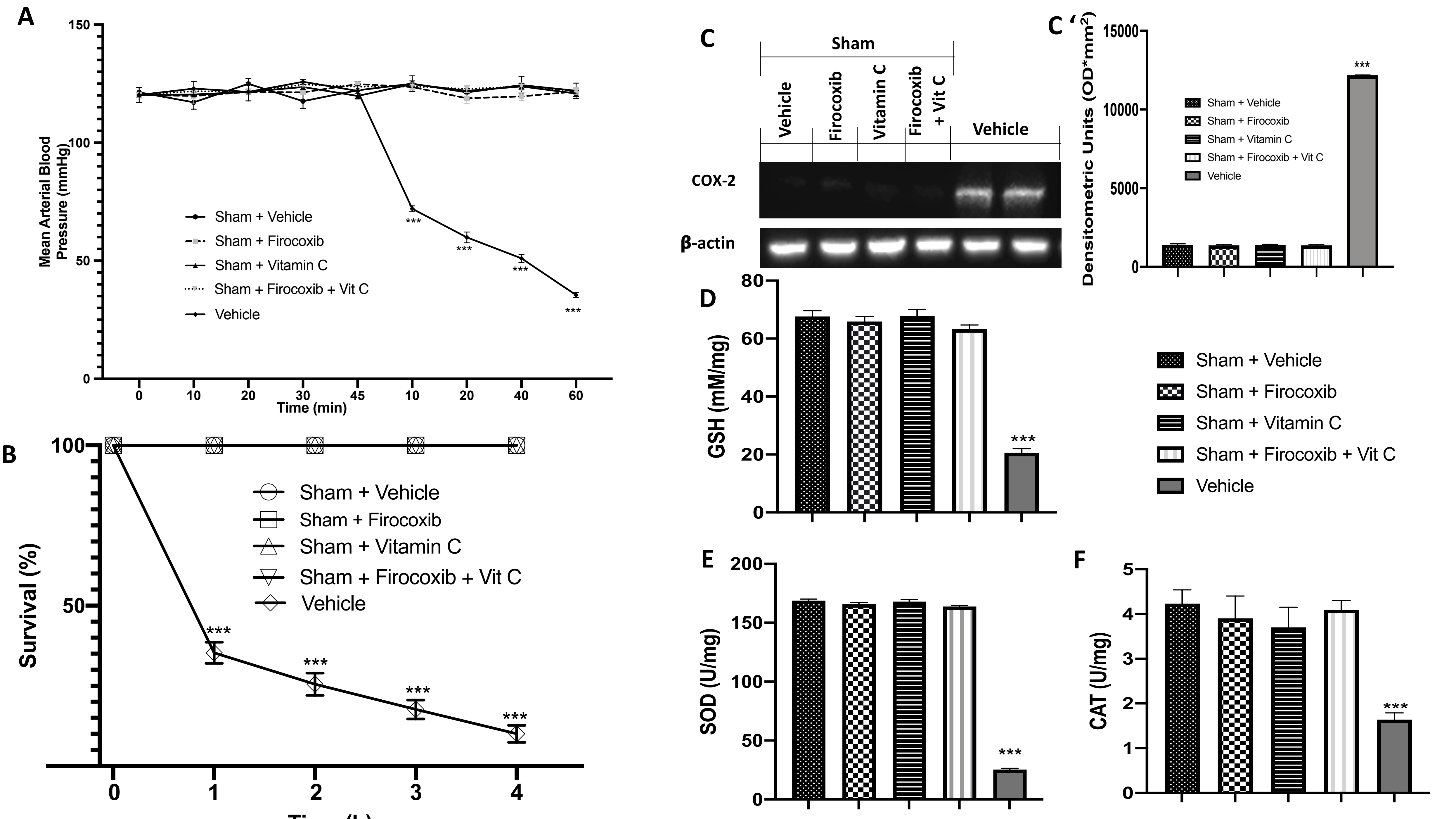
A
Mean Arterial Blood Pressure (mmHg)
150
100
50
0
0
10
20
30
45
10
20
40
60
Time (min)
Sham + Vehicle
Sham + Firocoxib
Sham + Vitamin C
Sham + Firocoxib + Vit C
Vehicle
***
B
Survival (%)
100
50
0
1
2
3
4
***
C
Sham
Vehicle
Firocoxib
Vitamin C
Firocoxib + Vit C
COX-2
β-actin
C ‘
Densitometric Units (OD*mm²)
15000
10000
5000
D
GSH (mM/mg)
80
60
40
20
E
SOD (U/mg)
200
150
100
F
CAT (U/mg)
5
4
3
2
1
